# Case mix, outcome and activity for obstetric admissions to adult, general critical care units: a secondary analysis of the ICNARC Case Mix Programme Database

**DOI:** 10.1186/cc3542

**Published:** 2005-06-17

**Authors:** David A Harrison, James A Penny, Steve M Yentis, Samia Fayek, Anthony R Brady

**Affiliations:** 1 Statistician, Intensive Care National Audit and Research Centre (ICNARC), London, UK; 2 Consultant Obstetrician and Gynaecologist, Surrey and Sussex Healthcare NHS Trust, East Surrey Hospital, Redhill, Surrey, UK; 3 Consultant Anaesthetist, Magill Department of Anaesthesia, Intensive Care and Pain Management, Chelsea and Westminster Hospital, London, UK; 4 Intensivist, Birmingham Heartlands and Solihull Hospitals, Birmingham, UK; 5 Senior Statistician, ICNARC, London, UK

## Abstract

**Introduction:**

Risk prediction scores usually overestimate mortality in obstetric populations because mortality rates in this group are considerably lower than in others. Studies examining this effect were generally small and did not distinguish between obstetric and nonobstetric pathologies. We evaluated the performance of the Acute Physiology and Chronic Health Evaluation (APACHE) II model in obstetric admissions to critical care units contributing to the ICNARC Case Mix Programme.

**Methods:**

All obstetric admissions were extracted from the ICNARC Case Mix Programme Database of 219,468 admissions to UK critical care units from 1995 to 2003 inclusive. Cases were divided into direct obstetric pathologies and indirect or coincidental pathologies, and compared with a control cohort of all women aged 16–50 years not included in the obstetric categories. The predictive ability of APACHE II was evaluated in the three groups. A prognostic model was developed for direct obstetric admissions to predict the risk for hospital mortality. A log-linear model was developed to predict the length of stay in the critical care unit.

**Results:**

A total of 1452 direct obstetric admissions were identified, the most common pathologies being haemorrhage and hypertensive disorders of pregnancy. There were 278 admissions identified as indirect or coincidental and 22,938 in the nonpregnant control cohort. Hospital mortality rates were 2.2%, 6.0% and 19.6% for the direct obstetric group, the indirect or coincidental group, and the control cohort, respectively. Cox regression calibration analysis showed a reasonable fit of the APACHE II model for the nonpregnant control cohort (slope = 1.1, intercept = -0.1). However, the APACHE II model vastly overestimated mortality for obstetric admissions (mortality ratio = 0.25). Risk prediction modelling demonstrated that the Glasgow Coma Scale score was the best discriminator between survival and death in obstetric admissions.

**Conclusion:**

This study confirms that APACHE II overestimates mortality in obstetric admissions to critical care units. This may be because of the physiological changes in pregnancy or the unique scoring profile of obstetric pathologies such as HELLP syndrome. It may be possible to recalibrate the APACHE II score for obstetric admissions or to devise an alternative score specifically for obstetric admissions.

## Introduction

Risk prediction scores, such as Acute Physiology and Chronic Health Evaluation (APACHE) II and III, and Simplified Acute Physiology Score II, are used to stratify the risk for death for each admission to a critical care unit in order to standardize data for the purposes of audit and research. They have also been modified for clinical use as early warning scores in general wards to help junior medical and nursing staff to identify those patients who are at risk for requiring medical attention or admission to an intensive care unit (ICU).

Several scores have been evaluated in obstetric patients in general ICUs and found to overestimate [1-4], underestimate [5] and accurately predict [6,7] mortality. These surveys were relatively small and retrospective and therefore may not have identified all suitable cases. In particular, not all distinguished between obstetric and nonobstetric pathologies.

It is known that mortality rates for obstetric admissions to ICUs are lower than those for the population background, particularly in women with obstetric pathologies such as severe pre-eclampsia and massive haemorrhage. Because the rate of obstetric admission to ICU is low, there is little opportunity for any individual to gain extensive clinical experience. Evaluating the APACHE II score in obstetric patients would facilitate the development of clinical care pathways, allow appropriate risk stratification and promote the development of a specific obstetric severity of illness score.

We evaluated the performance of the APACHE II score for the prediction of mortality in women with primary obstetric pathologies and those with coincidental pathologies while pregnant, using a high-quality clinical database of admissions to general critical care units. Secondary analysis was performed to develop a revised model for the prediction of mortality and length of stay.

## Materials and methods

### Case Mix Programme Database

The Case Mix Programme (CMP) is a national comparative audit of adult, general critical care units (including ICUs and combined intensive care and high dependency units) in England, Wales and Northern Ireland, co-ordinated by the Intensive Care National Audit and Research Centre (ICNARC). Data were extracted for 219,468 admissions from 159 critical care units from the CMP Database (CMPD), covering the period from December 1995 to June 2003 inclusive. Details regarding data collection and validation were reported previously [8].

### Selection of cases

Details regarding admissions of females aged 16–50 years inclusive were selected from the CMPD. Obstetric admissions were identified from the 'Primary reason for admission' and 'Secondary reason for admission' fields, and from either of two, optionally recorded 'Other condition relevant to the admission' fields. These four fields are all coded using the ICNARC Coding Method [9] – a hierarchical method specifically designed for coding reasons for admission to ICU. Additional cases were identified by searching the free text field of the database. All admissions identified from the text field search and not from other fields were checked by one author for appropriateness. When there was uncertainty regarding whether such a case should be included, a consensus was arrived at among all authors.

Two groups of obstetric admissions were identified, namely direct obstetric admissions and indirect or coincidental obstetric admissions. Direct obstetric admissions included all women for whom the 'Primary reason for admission' or 'Secondary reason for admission' field contained any condition from Table 1. Indirect or coincidental obstetric admissions included all women who did not fall into the direct obstetric admission category and met any of the following criteria: the 'Other condition relevant to the admission' fields contained any condition from Table 1; the entry in the 'Primary reason for admission', 'Secondary reason for admission', or 'Other condition relevant to the admission' fields was any partially completed code with the site tier recorded as 'Ovary, fallopian tubes, uterus or genitalia (obstetric)'; or the patient was identified as being pregnant or having recently been pregnant by searching the text field for a predefined list of pregnancy-related search terms.

**Table 1:** Obstetric conditions in the ICNARC Coding Method

Haemorrhage	Hypertensive disorder	Other Conditions
Antepartum haemorrhage	HELLP syndrome	Ectopic pregnancy
Peripartum or postpartum haemorrhage	Pre-eclampsia	Amnionitis
	Eclampsia	Infected retained products of conception
		Septic abortion
		Intrauterine death
		Molar pregnancy
		Amniotic fluid embolus

HELLP, haemolysis, elevated liver enzymes and low platelets; ICNARC, Intensive Care National Audit and Research Centre.

The term 'indirect or coincidental' was chosen to reflect the fact that current or recent pregnancy may or may not have influenced the patient's requirement for intensive care or the decision to admit her to the critical care unit. The phrase 'all obstetric admissions' is used to refer to all admissions identified as either a direct obstetric admission or an indirect or coincidental obstetric admission, following the methods described above.

The remaining group of admissions of females aged 16–50 years who were not identified as a direct obstetric admission or an indirect or coincidental obstetric admission was used as a comparison population.

### Data

Data were extracted regarding case mix, outcome and activity as defined below.

#### Case mix

Severity of illness was measured using the APACHE II Acute Physiology Score (APS) and the APACHE II score [10]. The APS encompasses a weighting for acute physiology (defined by derangement from the normal range for 12 physiological variables within the first 24 hours following admission to ICU). The APACHE II score also encompasses a weighting for age and for a past medical history of specified serious comorbidities.

Surgical status was defined as either nonsurgical, elective surgery, or emergency surgery, based on the source of admission to the CMP unit and the National Confidential Enquiry into Perioperative Death classification of surgery, as was described previously [8].

#### Outcome

Survival data were extracted at discharge from the CMP unit and at ultimate discharge from hospital.

#### Activity

Length of stay in the CMP unit was calculated as a fraction of days from the dates and times of admission and discharge. Length of stay in hospital was calculated in days from the dates of original admission and ultimate discharge. Readmissions to the unit within the same hospital stay were identified from the postcode, date of birth and sex, and confirmed by the participating units.

### Analyses

A statistical analysis plan was agreed *a priori*. The analyses performed were as follows.

#### Descriptive statistics

Case mix, outcome and activity were described for each of the four defined groups: direct obstetric admissions (group 1); indirect or coincidental obstetric admissions (group 2); all obstetric admissions (group 3; includes all women in groups 1 and 2); and female nonobstetric admissions aged 16–50 years (group 4). Numbers of admissions and ultimate hospital mortality rates were reported for each individual obstetric condition. The most common primary reasons for admission to the critical care unit (conditions accounting for five or more admissions) were also reported for indirect or coincidental obstetric admissions.

#### Evaluation of APACHE II score in obstetric admissions

The prognostic ability of the APACHE II model [10] was assessed in the four defined groups. The APACHE II score was evaluated for discrimination (the ability of the model to distinguish survivors from nonsurvivors), and the APACHE II mortality probability (using coefficients from the UK model [11]) was evaluated for discrimination and calibration (the accuracy of the estimated probability of survival). Discrimination was assessed by the area under the receiver operating characteristic (ROC) curve (AUC) [12], and calibration by the mortality ratio (observed over expected deaths), the Hosmer–Lemeshow C statistic (based on 10 equally sized groups) [13] and Cox regression calibration [14].

Cox regression calibration tests for a systematic lack of calibration by performing a linear recalibration of the log odds. The following model is fitted: observed log odds = slope × predicted log odds + intercept. If the model is perfectly calibrated then the slope will be 1 and the intercept 0 (i.e. observed log odds = predicted log odds). The result of the Cox calibration model also provides a simple method by which to recalibrate a poorly calibrated model.

Admissions of females who stayed less than 8 hours in the critical care unit were excluded from the calculation of APACHE II scores and probabilities, as were readmissions within the same hospital stay, transfers from another critical care unit and admissions following coronary artery bypass graft or for primary burns. These represent the standard exclusions for APACHE II.

#### Prognostic modelling in obstetric admissions for haemorrhage or hypertensive disorders

The effect of case mix factors on ultimate hospital mortality was assessed by multiple logistic regression modelling in all direct obstetric admissions with a primary or secondary reason for admission of antepartum, peripartum, or postpartum haemorrhage, and those with a hypertensive disorder of pregnancy (haemolysis, elevated liver enzymes and low platelets [HELLP] syndrome, or pre-eclampsia or eclampsia). These subsets of obstetric admissions were selected for modelling because they each represent distinct, large and relatively homogenous groups of obstetric admissions, and together they account for the majority of direct obstetric admissions.

The variables entered into the model, selected *a priori*, were as follows: age; reason for admission (haemorrhage or hypertensive disorder); surgical status; presence of any APACHE II chronic health condition; highest central temperature (or highest noncentral temperature +1°C if no central temperature recorded); extreme systolic blood pressure (furthest from the normal range of 100–170 mmHg); extreme heart rate (furthest from the normal range of 60–100 beats/min); highest respiratory rate; arterial oxygen tension (PaO_2_)/fractional inspired oxygen ratio from the arterial blood gas with the lowest PaO_2_; lowest pH; lowest serum sodium; extreme serum potassium (furthest from the normal range of 3.0–5.5 mmol/l); highest serum creatinine; highest serum urea; highest total serum bilirubin; extreme haematocrit (furthest from the normal range of 32–38%); lowest white blood cell count; lowest platelet count; and lowest total Glasgow Coma Scale (GCS) score (or presedation GCS score for admissions sedated or paralyzed for entire first 24-hour period in the CMP unit).

All physiological variables, with the exception of presedation GCS score, were recorded as the most extreme measurement during the first 24 hours in the CMP unit. In the event of death during the first 24 hours in the CMP unit, data were considered valid up to the earliest documented time of decision to withdraw all active treatment, certification of brainstem death, or certification of death, with agonal values valid only if charted. All continuous variables were modelled as having a linear effect on the log odds. Each variable was screened for missing values; those variables with more than 30% of values missing were dropped from the analysis to restrict the model to include only routinely measured variables. Missing values for the remaining variables were imputed to the median value. These variables were entered into a multiple logistic regression model, with the least significant variable being removed in a stepwise manner until no variables remained. At each step the discrimination of the model was evaluated by the AUC. The best model was selected by using the Akaike Information Criterion [15], which has been shown to be an appropriate method for selecting the degree to which a model should be simplified [16].

#### Modelling length of stay in the intensive care unit in obstetric admissions for haemorrhage or hypertensive disorders

The effect of case mix factors on length of stay in the CMP unit was explored in the same admissions as for the prognostic modelling. The same variables as were used in prognostic modelling were entered into a linear regression model on the logarithm of length of stay. No stepwise selection was performed, with results presented from the full model.

#### Statistical analysis

All analyses were performed using Stata 8.2 (Stata Corporation, College Station, TX, USA). *P *< 0.05 was considered statistically significant.

## Results

### Data

**Figure 1:**
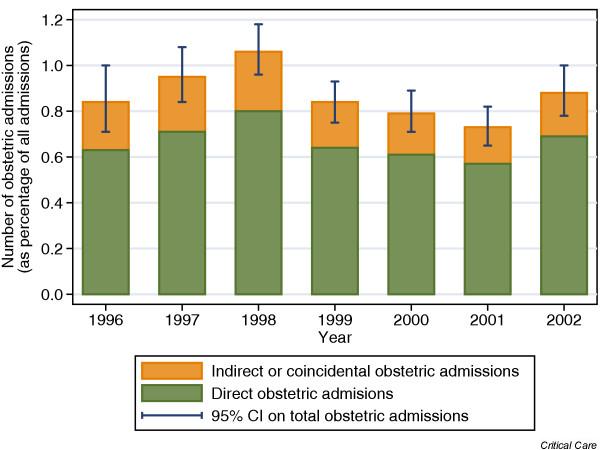
Trend in obstetric admissions (1996–2002). CI, confidence interval.

Of 219,468 admissions in the CMPD, 1452 (0.7%) were identified as direct obstetric admissions. A further 278 admissions were identified as indirect or coincidental obstetric admissions by the presence of an obstetric code in the 'Other conditions relevant to the admission' or a partially completed obstetric code in any field. Additionally, 175 admissions matched one or more of the terms used in the text field search. On inspection by one of us (DH), 164 clearly met the condition of 'being pregnant or having recently been pregnant' and the remaining 11 were discussed among the authors, with eight being included and three excluded once consensus was reached. This left a total of 450 indirect or coincidental obstetric admissions (0.2% of all CMPD admissions). The comparison group of all nonobstetric female admissions aged 16–50 years consisted of 22,938 admissions (10.5% of all CMPD admissions). In total, the 1902 obstetric admissions represented 0.9% of all CMPD admissions and 7.7% of all female admissions aged 16–50 years. The trend in obstetric admissions over time (as a percentage of all admissions) for the 7 complete years from 1996 to 2002 inclusive is shown in Fig. 1. After adjusting for the changing units participating in the CMP, there was no significant trend over time (odds ratio = 0.96 per year, 95% confidence interval [CI] 0.94–1.01).

### Descriptive statistics

The case mix, outcome and activity of these groups of admissions are summarized in Table 2.

**Table 2:** Case mix, outcome and activity for obstetric and non-obstetric admissions to critical care units

	All obstetric admissions (*n *= 1902)	Direct obstetric admissions (*n *= 1452)	Indirect or coincidental obstetric admissions (*n *= 450)	Female nonobstetric admissions aged 16–50 years (*n *= 22,938)
Case mix				
Age (years)^a^	30 (25–34) [16–50]	30 (25–34) [16–47]	29 (24–34) [16–50]	37 (28–44) [16–50]
APACHE II APS^b,d^	10.8 (5.5) [0–41]	10.7 (5.3) [0–41]	11.2 (5.8) [0–34]	12.7 (6.9) [0–50]
APACHE II score^d^	10.9 (5.5) [0–41]	10.8 (5.4) [0–41]	11.4 (6.0) [2–39]	13.7 (7.3) [0–50]
Surgical status^c^				
Nonsurgical	862 (45.4)	630 (43.4)	232 (51.6)	16,246 (70.9)
Elective surgery	145 (7.6)	75 (5.2)	70 (15.5)	3550 (15.5)
Emergency surgery	893 (47.0)	745 (51.4)	148 (32.9)	3123 (13.6)
Outcome				
Mortality^c^				
CMP unit (ICU)	44 (2.3)	25 (1.7)	19 (4.2)	3372 (14.7)
Any hospital^e^	58 (3.1)	32 (2.2)	26 (6.0)	4206 (19.6)
Activity				
Length of stay (days)				
CMP unit (survivors)	1.1 (0.7–2.3) [0–72]	1.1 (0.7–2.1) [0–41]	1.1 (0.6–2.7) [0–72]	1.5 (0.8–3.8) [0–209]
CMP unit (nonsurvivors)	1.3 (0.5–4.5) [0–59]	1.1 (0.3–10.3) [0–59]	1.5 (0.6–3.2) [0–21]	1.8 (0.7–5.2) [0–165]
Any hospital^e ^(survivors)	10 (6–15) [0–417]	9 (6–14) [1–417]	11 (6–20) [0–373]	12 (6–27) [0–767]
Any hospital^e ^(nonsurvivors)	4.5 (2–13) [0–79]	6 (1.5–14) [0–62]	3.5 (2–10) [0–79]	5 (2–16) [0–669]
Readmissions^c^	17 (0.9)	7 (0.5)	10 (2.2)	945 (4.1)

Values are expressed as ^a^median (interquartile range) [range], ^b^mean (standard deviation) [range], or ^c^*n *(%). ^d^Acute Physiology and Chronic Health Evaluation (APACHE) II exclusions: age <16 years, stay <8 hours, readmission within same hospital stay, transfer from another intensive care unit (ICU), admission following coronary artery bypass grafting, and admission for primary burns. ^e^Excluding readmissions within the same hospital stay. APS, Acute Physiology Score; CMP, Case Mix Programme.

The median age of obstetric admissions was 30 years, and this was similar for direct obstetric admissions and indirect or coincidental obstetric admissions. The mean APACHE II score was 10.9, which was very similar to the APS. This is to be expected because age points are only assigned above 45 years and the very severe chronic health conditions of APACHE II are rare in this age group. Most admissions were either following emergency surgery (47%) or nonsurgical (45%), with very few admissions following elective surgery (8%).

**Figure 2:**
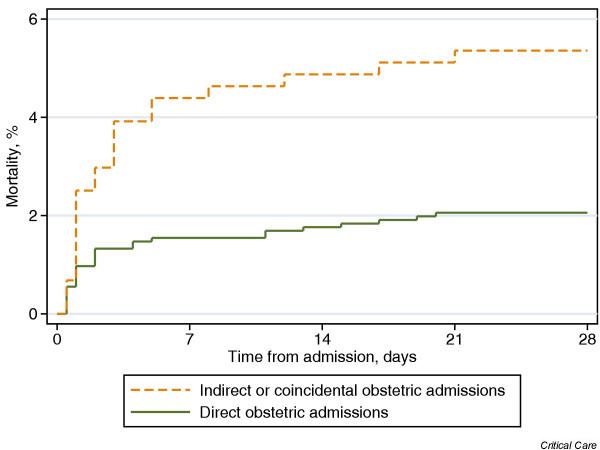
Twenty-eight-day Kaplan–Meier mortality plots for direct and indirect or coincidental obstetric admissions. Admissions discharged alive from hospital before 28 days are assumed to survive to at least 28 days.

Mortality among obstetric admissions was very low, with only 2.2% of patients with direct obstetric admissions dying before ultimate discharge from hospital, versus 6.0% among indirect or coincidental obstetric admissions and 19.6% among female nonobstetric admissions aged 16–50 years (χ^2 ^test, *P *< 0.001). A Kaplan–Meier plot of mortality during the 28 days following admission to the CMP unit is shown in Fig. 2. In total, 14 of those with obstetric admissions died in hospital after discharge from the CMP unit. One of these patients was discharged for palliative care. Of the remaining 13 patients, nine were transferred to critical care units in the same or another hospital of whom six died in the subsequent critical care unit, two were discharged to a ward in the same hospital of whom one was subsequently readmitted to and died in the original unit, and two were transferred to another hospital.

The median length of stay for survivors in the CMP unit was 1.1 days for both direct and indirect or coincidental obstetric admissions. This was slightly shorter than for those with nonobstetric admissions, who stayed a median of 1.5 days (Wilcoxon rank-sum test, *P *< 0.001).

Table 3 shows the numbers of admissions with each specific individual obstetric condition identified in the ICNARC Coding Method and the mortality within each condition. The most common condition was peripartum or postpartum haemorrhage, accounting for 29% of all obstetric admissions or 0.25% of all admissions in the CMPD.

**Table 3:** Prevalence of obstetric conditions in any of the four ICNARC Coding Method fields in the CMPD

ICNARC Coding Method condition	*n*	% of all obstetric admissions	Ultimate hospital mortality (*n *[%])
Peripartum or postpartum haemorrhage	553	29.1	3 (0.6)
Pre-eclampsia	347	18.2	7 (2.0)
HELLP syndrome	239	12.6	6 (2.6)
Eclampsia	141	7.4	5 (3.5)
Ectopic pregnancy	104	5.5	1 (1.0)
Intrauterine death	95	5.0	6 (6.3)
Antepartum haemorrhage	71	3.7	5 (7.2)
Infected retained products of conception	26	1.4	1 (3.8)
Amniotic fluid embolus	22	1.2	2 (9.1)
Septic abortion	18	0.9	2 (11.1)
Amnionitis	7	0.4	1 (16.7)
Molar pregnancy	4	0.2	1 (25.0)
Any obstetric condition	1496	78.7^a^	37 (2.5)

Note that the columns do not sum to the values in the 'Any obstetric condition' row because some admissions had more than one obstetric condition recorded in the four fields. ^a^The remaining 406 obstetric admissions (21.3%) were identified from a partial obstetric code (234) or by the text field search (172). CMPD, Case Mix Programme Database; HELLP, haemolysis, elevated liver enzymes and low platelets; ICNARC, Intensive Care National Audit and Research Centre.

Table 4 summarizes the primary reasons for admission accounting for five or more indirect or coincidental obstetric admissions. The most common primary reason for admission was status epilepticus or uncontrolled seizures, accounting for 19 admissions (4.2% of all indirect or coincidental obstetric admissions). Overall, 115 different conditions were reported as primary reasons for admission among these 450 admissions.

**Table 4:** Most common primary reasons for admission to the critical care unit for indirect or coincidental obstetric admissions

ICNARC Coding Method condition	*n*	% of indirect obstetric admissions
Status epilepticus or uncontrolled seizures	19	4.2
Asthma attack in new or known asthmatic	16	3.6
Septic shock	16	3.6
Pneumonia, no organism isolated	15	3.3
Noncardiogenic pulmonary oedema (ARDS)	11	2.4
Anaphylaxis	10	2.2
Cardiogenic pulmonary oedema	10	2.2
Pulmonary embolus (thrombus)	10	2.2
Septicaemia	9	2.0
Bacterial pneumonia	8	1.8
Acute renal failure, other cause^a^	7	1.6
Hypovolaemic shock	7	1.6
Pelvic infection or abscess	7	1.6
Acute pancreatitis	6	1.3
Epidural injection or infusion	6	1.3
Appendicitis or appendix abscess	5	1.3
Intracerebral bleeding	5	1.1
Phaeochromocytoma	5	1.1
Spinal injection or infusion	5	1.1
Supraventricular tachycardia, atrial fibrillation or flutter	5	1.1
Toxic or drug-induced coma or encephalopathy	5	1.1
Viral pneumonia	5	1.1

Conditions accounting for five or more indirect or coincidental obstetric admissions. ^a^Not due to infection, haemodynamic, toxic, or drug causes. ARDS, acute respiratory distress syndrome.

### Evaluation of APACHE II in obstetric admissions

**Figure 3:**
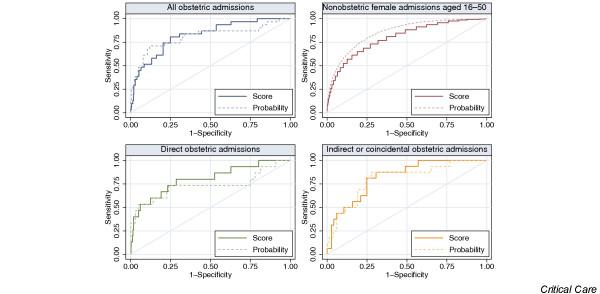
ROC curves for APACHE II score and mortality probability in obstetric and nonobstetric admissions. APACHE, Acute Physiology and Chronic Health Evaluation; ROC, receiver operating characteristic.

**Figure 4:**
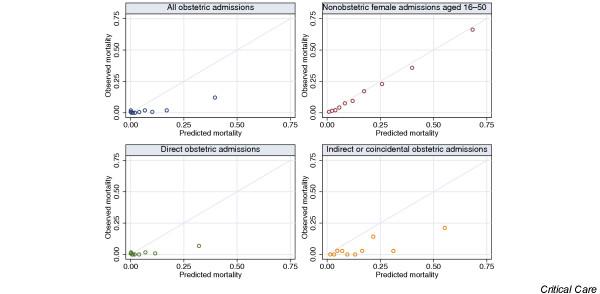
Calibration plots for APACHE II mortality probability in obstetric and nonobstetric admissions. Observed mortality is plotted against deciles of predicted mortality. Diagonal line indicates perfect calibration. APACHE, Acute Physiology and Chronic Health Evaluation.

Measures of discrimination and calibration for the APACHE II model are given in Table 5. Plots of the ROC curves and calibration plots are shown in Figs 3 and 4, respectively.

**Table 5:** Measures of discrimination and calibration for APACHE II in obstetric and non-obstetric admissions

	All obstetric admissions (*n *= 1565)	Direct obstetric admissions (*n *= 1216)	Indirect or coincidental obstetric admissions (*n *= 349)	Female nonobstetric admissions aged 16–50 years (*n *= 18,450)
Discrimination (AUC [95% CI])				
APACHE II score^a^	0.839 (0.820 to 0.857)	0.819 (0.795 to 0.840)	0.839 (0.797 to 0.876)	0.812 (0.807 to 0.818)
APACHE II probability^a^	0.810 (0.790 to 0.829)	0.737 (0.711 to 0.761)	0.806 (0.760 to 0.845)	0.855 (0.850 to 0.860)
Calibration				
Mortality ratio (95% CI)	0.245 (0.167 to 0.346)	0.214 (0.120 to 0.352)	0.282 (0.163 to 0.453)	0.907 (0.878 to 0.937)
Hosmer–Lemeshow				
C statistic (*P *value)	166 (< 0.001)	101 (<0.001)	47 (<0.001)	69 (<0.001)
Cox's regression calibration				
Slope (95% CI)	0.728 (0.491 to 0.966)	0.570 (0.277 to 0.863)	0.937 (0.509 to 1.37)	1.05 (1.01 to 1.08)
Intercept (95% CI)	-2.09 (-2.60 to -1.59)	-2.57 (-3.39 to -1.76)	-1.69 (-2.33 to -1.05)	-0.120 (-0.179 to -0.061)
χ^2^_(2) _(*P *value)^a^	140 (<0.001)	87 (<0.001)	55 (<0.001)	57 (<0.001)

^a^χ^2 ^statistic with two degrees of freedom (and associated *P *value) from a likelihood ratio test of slope 1 and intercept 0 in Cox's regression calibration model. APACHE, Acute Physiology and Chronic Health Evaluation; AUC, area under the receiver operating characteristic curve; CI, confidence interval.

Discrimination of the APACHE II score was good in all groups, and better in obstetric admissions (AUC = 0.839, 95% CI = 0.820–0.857) than in the control cohort of female nonobstetric admissions aged 16–50 years (AUC = 0.812, 95% = CI 0.807–0.818). In obstetric admissions, using the APACHE II mortality probability in place of the score did not improve discrimination.

**Figure 5:**
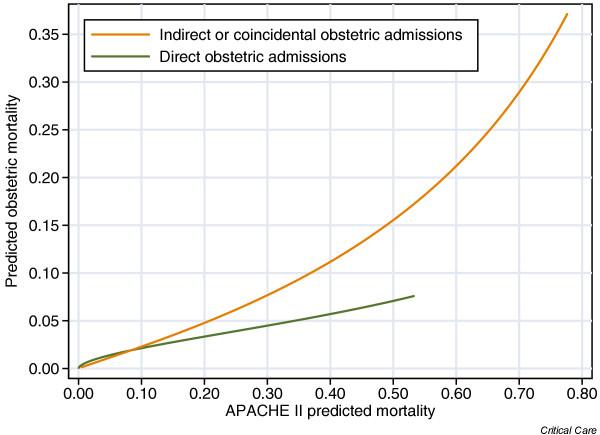
Recalibration of the APACHE II predicted mortality in obstetric admissions. Lines show the results of recalibration by Cox regression calibration (linear recalibration of the log odds). Lines are plotted between the 1st and 99th percentiles of observed APACHE II mortality predictions. APACHE, Acute Physiology and Chronic Health Evaluation.

Calibration of the APACHE II mortality probability was extremely poor in obstetric admissions (mortality ratio 0.245 for all obstetric admissions versus 0.907 for the control cohort). Although the Hosmer–Lemeshow C statistic and Cox regression calibration showed statistically significant departures from perfect calibration in the control cohort, the calibration plot (Fig. 4) shows that the prediction in this group was qualitatively quite good compared with the obstetric admissions, in which the mortality was vastly overestimated. Calibration was poor in both the direct and indirect or coincidental subgroups of obstetric admissions. Figure 5 shows the recalibration of the APACHE II mortality probability from the Cox regression calibration model for direct and indirect or coincidental obstetric admissions. This provides a simple method with which to adjust the predicted mortality in these groups; for example, a direct obstetric admission with an APACHE II mortality probability of 0.3 would have a predicted mortality of just under 0.05 according to this model. The lines are drawn only from the 1st to the 99th percentiles of the observed APACHE II mortality predictions, because extrapolation outside of this range may be unsafe.

### Prognostic modelling in obstetric admissions for haemorrhage or hypertensive disorders

A total of 1232 admissions met inclusion criteria (primary or secondary reason for admission of haemorrhage or hypertensive disorder) for modelling mortality and length of stay. The variables total serum bilirubin, serum albumin and haematocrit were dropped from the models because they were missing in more than 30% of all eligible admissions, leaving 17 variables in the initial full model. A further 20 admissions were missing ultimate hospital mortality status, and so the mortality model was fitted on 1212 admissions.

**Figure 6:**
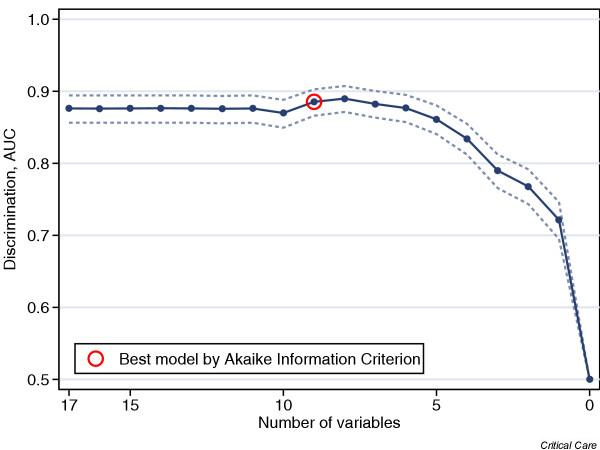
Plot of AUC for ROC curve against variables for the multiple logistic regression models. The best model, as selected using the Akaike Information Criterion, is indicated. AUC, area under the curve; ROC, receiver operating characteristic.

Figure 6 shows the AUC for each model in the stepwise selection procedure with the best model, as selected using the Akaike Information Criterion, identified. The odds ratio estimates from the best model are shown in Table 6. Nine variables remained in the best model, and of these four were significant at the 5% level (identified in Table 6). The best model had an AUC of 0.885 (95% CI = 0.866–0.903).

**Table 6:** Odds ratio estimates from the best logistic regression model as selected by the Akaike Information Criterion (*n *= 1212)

Variable	Deaths	*n *(%)		Odds ratio (95% CI)	*P*
Type of condition					
Haemorrhage	6	581 (1.0)		1.00	0.167
Hypertensive	16	631 (2.5)		2.30 (0.71–7.49)	
Surgical status					
Surgical (admission from theatre)	6	662 (0.9)		1.00	0.093
Nonsurgical	16	550 (2.9)		2.53 (0.86–7.44)	
Past medical history present*^a^					
No	20	1200 (1.7)		1.00	0.010
Yes	2	12 (16.7)		14.91 (1.88–118.13)	
SBP (mmHg)*					
Below normal (<100)	129	338 (3.6)		1.38 (1.06–1.81) per 10 mmHg deviation from normal	0.017
Normal range (100–170)	1	467 (0.2)			
Above normal (>170)	9	407 (2.2)			
Heart rate (beats/min)*					
Below normal (<60)	1	86 (1.2)		1.36 (1.08–1.71) per 10 beats/min deviation from normal	0.009
Normal range (60–100)	1	248 (0.4)			
Above normal (>100)	20	878 (2.3)			
PaO_2_/FiO_2 _ratio (mmHg)					
<280	14	409 (3.4)		1.49 (0.98–2.27) per 100 mmHg decrease	0.063
280–330	4	413 (1.0)			
>330	4	390 (1.0)			
Potassium (mmol/l)					
Below normal (<3)	2	29 (6.9)		0.18 (0.01 – 2.46) per 1 mmol/l deviation from normal	0.198
Normal range (3–5.5)	18	1069 (1.7)			
Above normal (>5.5)	2	114 (1.8)			
Lowest WBC (× 10^9^/l)					
<10	8	409 (2.0)		1.67 (0.96–2.92) per 5 × 10^9^/l decrease	0.070
10–13	7	357 (2.0)			
>13	7	446 (1.6)			
GCS*					
3–10	10	86	(11.6)	1.27 (1.16–1.39) per 1 point decrease	<0.001
11–14	2	94	(2.1)		
15	10	1032	(1.0)		

^a^Evidence of any of a prespecified list of severe conditions in the past medical history, as defined in Acute Physiology and Chronic Health Evaluation (APACHE) II. CI, confidence interval; GCS, Glasgow Coma Scale; PaO_2_/FiO_2_, arterial oxygen tension; fractional inspired oxygen; SBP, systolic blood pressure; WBC, white blood count. *P < 0.05.

The strongest predictor was GCS score. The model with this variable alone had an AUC of 0.721 (95% CI = 0.695–0.746).

### Modelling length of stay in the Case Mix Programme unit in obstetric admissions for haemorrhage or hypertensive disorders

Information on date or time of discharge from the unit was missing for seven admissions, and so these were dropped from the analysis of length of stay. The length of stay model was fitted on a total of 1225 admissions.

The results of the multiple regression on length of stay in the CMP unit are presented in Table 7. The following were associated with an increased length of stay: hypertensive disorder, nonsurgical admission, higher temperature, deviation from normal systolic blood pressure, higher heart rate, lower PaO_2_/fractional inspired oxygen ratio, higher creatinine and lower white blood cell count. Lower sodium was associated with reduced length of stay.

**Table 7:** Effect estimates from the model of length of stay in the CMP unit (*n *= 1225)

Variable	Multiplicative effect on LOS (95% CI)	*P*
Type of condition*		
Haemorrhage	1.00	<0.001
Hypertensive	1.44 (1.29–1.61)	
Age (per 10-year increase)	1.06 (0.98–1.14)	0.165
Surgical status*		
Surgical (admission from theatre)	1.00	0.036
Nonsurgical	1.12 (1.01–1.24)	
Past medical history present^a^	1.04 (0.65–1.64)	0.883
Highest temperature (per 1°C increase)*	1.13 (1.06–1.20)	<0.001
SBP (per 10 mmHg deviation from normal)*	1.09 (1.05–1.13)	<0.001
Highest heart rate (per 10 beats/min increase)*	1.09 (1.06–1.12)	<0.001
Highest respiratory rate (per 10 breaths/min) increase	0.98 (0.91–1.06)	0.664
PaO_2_/FiO_2 _ratio (per 100 mmHg decrease)*	1.14 (1.09–1.19)	<0.001
Lowest pH (per 0.1 decrease)*	1.14 (1.06–1.23)	<0.001
Lowest sodium (per 5 mmol/l decrease)*	0.90 (0.84–0.97)	0.006
Potassium (per 1 mmol/l deviation from normal)	1.12 (0.94–1.33)	0.190
Highest creatinine (per 100 μmol/l increase)*	1.32 (1.20–1.45)	<0.001
Highest urea (per 5 mmol/l increase)	1.02 (0.94–1.11)	0.662
Lowest WBC (per 5 × 10^9^/l decrease)*	1.12 (1.06–1.17)	<0.001
Lowest platelet count (per 50 × 10^9^/l decrease)	1.01 (0.97–1.05)	0.563
GCS (per 1 point decrease)	1.01 (0.99–1.03)	0.370

^a^Evidence of any of a pre-specified list of severe conditions in the past medical history as defined in Acute Physiology and Chronic Health Evaluation (APACHE) II. CI, confidence interval; CPM, Case Mix Programme; GCS, Glasgow Coma Scale; LOS, length of stay; PaO_2_/FiO_2_, arterial oxygen tension; fractional inspired oxygen; SBP, systolic blood pressure; WBC, white blood count. *P < 0.05.

The model did not change in any qualitative way (the set of significant variables was identical and the effect sizes were similar in these variables) when the model was restricted to only those who were admitted and discharged alive and 'fully ready for discharge'.

## Discussion

This study is the largest analysis that has been undertaken of obstetric admissions to critical care units. The data provided confirm many previously published findings but also allowed an analysis of the case mix data to evaluate relative performance of each physiological variable in the prediction of maternal death.

The previously published largest obstetric dataset from the UK was that reported by Hazelgrove and coworkers [17], who reported an analysis of the South West Thames database, which collects data on admissions to 14 general critical care units. They identified 1.8% of all admissions (210 out of 11,385 cases) as related to pregnancy, as compared with 0.9% in the CMPD. In the South West Thames dataset, over a quarter of the admissions were identified by the 'responsible consultant', which was not possible in the CMPD. However, Hazelgrove and coworkers still identified 1.3% of all admissions as being obstetric from the APACHE diagnostic code.

The mortality rates were very similar in the two datasets (3.1% in CMPD versus 3.3% in South West Thames), as were the primary reasons for admission, the most common being hypertensive disorders of pregnancy and massive haemorrhage. The mortality rate was also similar to that in other, smaller studies in the UK [18-20]. However, small studies from other countries reported widely varying mortality rates, reflecting different spectra of disease and admission criteria and making comparisons across health care systems difficult [3,4,6,21-29].

Length of stay in the critical care unit was shorter for obstetric than for nonobstetric admissions (median length of stay for survivors 1.1 versus 1.5 days). This was similar to that in the South West Thames dataset, in which the overall median length of stay for obstetric admissions was reported to be 1 day. The length of stay for all groups was slightly shorter than for all ICU admissions overall (median length of stay for survivors 1.7 days) [8].

In previous studies, most risk prediction scores tended to overestimate mortality in obstetric pathologies [1-4,30,31]. This is likely to be, in part, due to the self-limiting nature of pre-eclampsia and haemorrhage (responsible for the majority of cases) if supportive management is provided. This occurs despite marked physiological variation from normal, and therefore high APACHE II scores. However, the lower score in the obstetric subset of the CMPD, compared with the nonpregnant female control cohort, suggests that either there is a lower threshold for admission of obstetric patients or that the score underestimates 'sickness' in obstetric patients who otherwise require admission. This latter explanation is contrary to the score overestimating mortality. It is possible that the APACHE II and other scores perform poorly in obstetric pathologies because of the physiological changes in pregnancy, or perhaps because of the unique scoring profile of the complications of pre-eclampsia, such as HELLP syndrome and eclampsia. These issues were recently considered by Gopalan and Muckart [32], who concluded that prediction of outcome based specifically on organ dysfunction might be a better option for scoring severity of illness in obstetric patients.

The ability of the APACHE II score to discriminate between survivors and nonsurvivors among women with obstetric pathology was better than for nonobstetric pathology, with an AUC of 0.839 versus 0.812. This figure is close to the original APACHE II validation of 0.863 in all critical care admissions [10]. However, the calibration of the model was extremely poor because of the low mortality. Our analysis suggests two possible approaches for improving upon the APACHE II model for women with obstetric pathology. The first is a simple recalibration, as indicated by the Cox regression calibration curve. The other approach is to use the revised probability model arrived at using stepwise regression analysis. It is not possible to validate either method prospectively on this dataset. Because of the low frequency of obstetric admissions, it was not practicable to withhold a random validation sample, even in such a large database as this.

The AUC from the revised probability model was very high (0.885). This may be optimistic due to the potential for overfitting, because it has been suggested that there should be at least 10 events (deaths) for each variable to be estimated in order for parameter estimates to be considered reliable [33]. In our best prognostic model, there were only 22 events from which to estimate nine variables, yielding approximately 2.4 events per variable. There is also the potential for overfitting because of interactions between the physiological variables, but we did not have sufficient data to be able to model such interactions. The Akaike Information Criterion [16] was used to select the best model because it has been shown to produce models with similar predictive performance to full models.

The most important predictor of mortality was the GCS score, although other variables did improve the model. It is interesting to note that the variables that were most predictive of mortality were not those that predicted length of stay. The modelling of the length of stay was used as a surrogate marker of 'sickness'. This outcome is subject to many other influences but the CMPD does not record therapeutic interventions or other outcome measures. The predictors of mortality may be those that measure preterminal events or possible irreversible pathology such as coma, which is uncommon in survivors, but it interesting to note the almost complete lack of concordance in those variables that predict death compared with those that predict length of stay other than systolic blood pressure and heart rate. It is therefore difficult to recommend selected measures that can be used in clinical practice to help with early warning scores that may predict admission to critical care units in obstetric patients.

One limitation of secondary analysis of data collected for another purpose is the lack of some known prognostic variables (e.g. weight, parity, gestation/time since delivery, ethnicity). Additionally, the identification of obstetric patients in the database was hampered by the lack of specific fields identifying those women admitted who were pregnant. The forthcoming revision of the CMP Dataset Specification will include new fields specifically relevant to obstetric admissions.

## Conclusion

This study demonstrates that, although the APACHE II score discriminates well between survivors and nonsurvivors, it is poorly calibrated in women with obstetric pathology because of the low mortality rate. It may be that a simple recalibration of the predicted mortality will accurately predict death, or that a revised scoring system could be developed for obstetric patients. The dramatically lower mortality of obstetric patients compared with general admissions may be due to a lower admission threshold, altered physiology producing a unique scoring profile, pregnancy identifying a group of patients with better underlying health status, or the self-limiting nature of most obstetric pathology if appropriate monitoring and supportive care can be provided.

## Key messages

• Overall, 0.7% of all admissions to UK ICUs were identified as being direct obstetric admissions.

• Mortality in the ICU was only 1.7% for these admissions, as compared with 4.2% for indirect or coincidental obstetric admissions and 14.7% for nonobstetric female admissions aged 16–50 years.

• The most common obstetric pathologies were haemorrhage and hypertensive disorders of pregnancy.

• The APACHE II model had good discrimination but it overestimated mortality.

• It may be possible to recalibrate the APACHE II model for obstetric admissions or to develop a new specific model.

## Abbreviations

APACHE = Acute Physiology and Chronic Health Evaluation; APS = Acute Physiology Score; AUC = area under the curve; CI = confidence interval; CMP = Case Mix Programme; CMPD = Case Mix Programme Database; GCS = Glasgow Coma Scale; HELLP = haemolysis, elevated liver enzymes and low platelets; ICNARC = Intensive Care National Audit and Research Centre; ICU = intensive care unit; PaO_2 _= arterial oxygen tension; ROC = receiver operating characteristic.

## Competing interests

The author(s) declare that they have no competing interests.

## Authors' contributions

DH performed the analyses. DH and JP drafted the manuscript. All authors contributed to the design and interpretation of the study and critical revision of the manuscript, and read and approved the final manuscript.
